# User Engagement, Acceptability, and Clinical Markers in a Digital Health Program for Nonalcoholic Fatty Liver Disease: Prospective, Single-Arm Feasibility Study

**DOI:** 10.2196/52576

**Published:** 2024-02-15

**Authors:** Sigridur Björnsdottir, Hildigunnur Ulfsdottir, Elias Freyr Gudmundsson, Kolbrun Sveinsdottir, Ari Pall Isberg, Bartosz Dobies, Gudlaug Erla Akerlie Magnusdottir, Thrudur Gunnarsdottir, Tekla Karlsdottir, Gudlaug Bjornsdottir, Sigurdur Sigurdsson, Saemundur Oddsson, Vilmundur Gudnason

**Affiliations:** 1 Department of Endocrinology, Metabolism and Diabetes Karolinska Institutet Stockholm Sweden; 2 Sidekick Health Kopavogur Iceland; 3 Icelandic Heart Association Kopavogur Iceland; 4 School of Health Sciences Faculty of Medicine University of Iceland Reykjavik Iceland

**Keywords:** digital health program, nonalcoholic fatty liver disease, NAFLD, cardiometabolic health, digital therapeutics, liver, chronic, hepatic, cardiometabolic, cardiovascular, cardiology, weight, acceptability, digital health, metabolic syndrome, diabetic, diabetes, diabetics, type 2, BMI, lifestyle, exercise, physical activity, coaching, diet, dietary, nutrition, nutritional, patient education, coach, feasibility, fat, body composition

## Abstract

**Background:**

Nonalcoholic fatty liver disease (NAFLD) has become the most common chronic liver disease in the world. Common comorbidities are central obesity, type 2 diabetes mellitus, dyslipidemia, and metabolic syndrome. Cardiovascular disease is the most common cause of death among people with NAFLD, and lifestyle changes can improve health outcomes.

**Objective:**

This study aims to explore the acceptability of a digital health program in terms of engagement, retention, and user satisfaction in addition to exploring changes in clinical outcomes, such as weight, cardiometabolic risk factors, and health-related quality of life.

**Methods:**

We conducted a prospective, open-label, single-arm, 12-week study including 38 individuals with either a BMI >30, metabolic syndrome, or type 2 diabetes mellitus and NAFLD screened by FibroScan. An NAFLD-specific digital health program focused on disease education, lowering carbohydrates in the diet, food logging, increasing activity level, reducing stress, and healthy lifestyle coaching was offered to participants. The coach provided weekly feedback on food logs and other in-app activities and opportunities for participants to ask questions. The coaching was active throughout the 12-week intervention period. The primary outcome was feasibility and acceptability of the 12-week program, assessed through patient engagement, retention, and satisfaction with the program. Secondary outcomes included changes in weight, liver fat, body composition, and other cardiometabolic clinical parameters at baseline and 12 weeks.

**Results:**

In total, 38 individuals were included in the study (median age 59.5, IQR 46.3-68.8 years; n=23, 61% female). Overall, 34 (89%) participants completed the program and 29 (76%) were active during the 12-week program period. The median satisfaction score was 6.3 (IQR 5.8-6.7) of 7. Mean weight loss was 3.5 (SD 3.7) kg (*P*<.001) or 3.2% (SD 3.4%), with a 2.2 (SD 2.7) kg reduction in fat mass (*P*<.001). Relative liver fat reduction was 19.4% (SD 23.9%). Systolic blood pressure was reduced by 6.0 (SD 13.5) mmHg (*P*=.009). The median reduction was 0.14 (IQR 0-0.47) mmol/L for triglyceride levels (*P*=.003), 3.2 (IQR 0.0-5.4) µU/ml for serum insulin (s-insulin) levels (*P*=.003), and 0.5 (IQR –0.7 to 3.8) mmol/mol for hemoglobin A_1c_ (HbA_1c_) levels (*P*=.03). Participants who were highly engaged (ie, who used the app at least 5 days per week) had greater weight loss and liver fat reduction.

**Conclusions:**

The 12-week-long digital health program was feasible for individuals with NAFLD, receiving high user engagement, retention, and satisfaction. Improved liver-specific and cardiometabolic health was observed, and more engaged participants showed greater improvements. This digital health program could provide a new tool to improve health outcomes in people with NAFLD.

**Trial Registration:**

Clinicaltrials.gov NCT05426382; https://clinicaltrials.gov/study/NCT05426382

## Introduction

Nonalcoholic fatty liver disease (NAFLD) is the most common chronic liver disease in the world [1]. NAFLD is defined as >5% fat in the liver (steatosis) among people who drink moderate amounts or no alcohol and have no other chronic liver diseases [2]. NAFLD reflects a spectrum of liver pathologies, ranging from simple steatosis to a more severe condition called nonalcoholic steatohepatitis (NASH), which includes inflammation and potential scarring of the liver [3]. The major comorbidities associated with NAFLD are central obesity, type 2 diabetes mellitus (DM), dyslipidemia, and metabolic syndrome [4]. The global prevalence of NAFLD is estimated to be 25% in the general population and the rising prevalence of NAFLD parallels that of obesity and type 2 DM, since NAFLD is a comorbidity in an estimated 55% of people with type 2 DM and in up to 80% of people with obesity [4-6]. Studies have shown that around 20% to 30% of people with NAFLD progress to NASH, with its consequent risks of liver scarring, cirrhosis, end-stage liver disease, and hepatocellular carcinoma [7]. Furthermore, NAFLD and NASH are associated with cardiovascular diseases, type 2 DM, and chronic kidney disease and pose a large burden on health care systems [8-10].

Growing evidence supports a common pathophysiological mechanism between metabolic syndrome and NAFLD and NASH, which often involves insulin resistance and dysfunctional adipose tissue [11]. Currently, no pharmacological treatment is approved for NAFLD or NASH, and, according to treatment guidelines, first-line therapy should focus on lifestyle improvement with the aim of 5% to 10% weight loss [12,13]. However, reaching these goals is often difficult, and there is a need to continue exploring optimal treatment modalities for individuals with NAFLD or NASH [14].

Sidekick Health, an Icelandic digital therapeutic company, has developed a digital health program (Sidekick-241 or SK-241) specifically designed for people with metabolic conditions and NAFLD. The 12-week program is delivered through a mobile app and aims to improve lifestyle and health outcomes by focusing on improving diet, increasing activity levels, and reducing stress through behavior change. In this prospective study, we evaluated the feasibility and potential clinical impact of the 12-week digital health program on liver and cardiometabolic health in individuals with metabolic conditions and NAFLD.

## Methods

### Trial Design

This was an open-label, single-arm, prospective study conducted between June and September 2022 in Iceland. The study included a 12-week digital health program delivered through the Sidekick app. Screening and preprogram and postprogram clinical assessments were carried out at the Icelandic Heart Association.

### Participants 

In total, 38 individuals aged between 18 and 80 years from an ongoing population-based cohort study (The REFINE-Reykjavik Study) at The Icelandic Heart Association and individuals followed at an endocrine outpatient clinic (the Reykjavik Heart Center) were invited to participate in the study [15]. People with at least one of the following risk factors were invited to participate: BMI >30, metabolic syndrome, or type 2 DM. Individuals with type 2 DM were only included if they were on a stable dose of antidiabetes medication for the last 90 days before screening. Eligible individuals had to have the capacity to give informed consent, understand verbal and written Icelandic, own and know how to operate a smartphone, and be willing and able to comply with the study program, all scheduled visits, and procedures.

The exclusion criteria were as follows: insulin use; known or self-reported cirrhosis; alcohol consumption over 14 units/week for men or over 7 units/week for women; self-reported hepatitis B, hepatitis C, human immunodeficiency virus, or autoimmune hepatitis; vitamin E intake of >400 IU/day unless stable for 12 weeks prior to baseline; taking medications associated with liver steatosis, such as steroids, methotrexate, tamoxifen, amiodarone, tetracycline, or valproic acid; self-reported pregnancy; participation in a weight loss program; or history of or any existing medical condition (eg, ongoing cancer treatment, severe cardiopulmonary or musculoskeletal disease); magnetic resonance imaging contraindications (eg, pacemakers, aneurysm clips), or stroke or myocardial infarction in the last 6 months that, in the opinion of the primary investigator, would interfere with evaluation of the study or affect the interpretation of the results of the study.

After obtaining informed consent, participants were screened for eligibility by study staff at the Icelandic Heart Association.

### Screening for NAFLD

Individuals were screened to assess if they had liver steatosis with a noninvasive ultrasonography-based controlled attenuation parameter (CAP) assessment through a FibroScan device [16]. To avoid overestimation of steatosis, we used 2 probe sizes (medium and extra-large). Individuals who met the full inclusion and exclusion criteria and had a CAP score of >294 dB/m, which represents a high likelihood of >5% liver steatosis, were eligible for participation in the study [17]. Additionally, liver stiffness measurement (LSM) was performed with vibration-controlled transient elastography (VCTE) at screening and at the 12-week follow-up visit. Individuals with an LSM score >9.7 kPa, which represents moderate-to-severe liver fibrosis (grade F3-F4), were referred to a specialist for further evaluation [18]. All individuals with a CAP score >294 dB/m had a magnetic resonance imaging proton density fat fraction (MRI-PDFF) measurement at screening and at the 12-week follow-up visit. MRI-PDFF is considered an emerging biomarker for non-invasive hepatic steatosis assessment as it is accurate, precise, quantitative, and reproducible [19].

### The Digital Health Program

**Figure 1:**
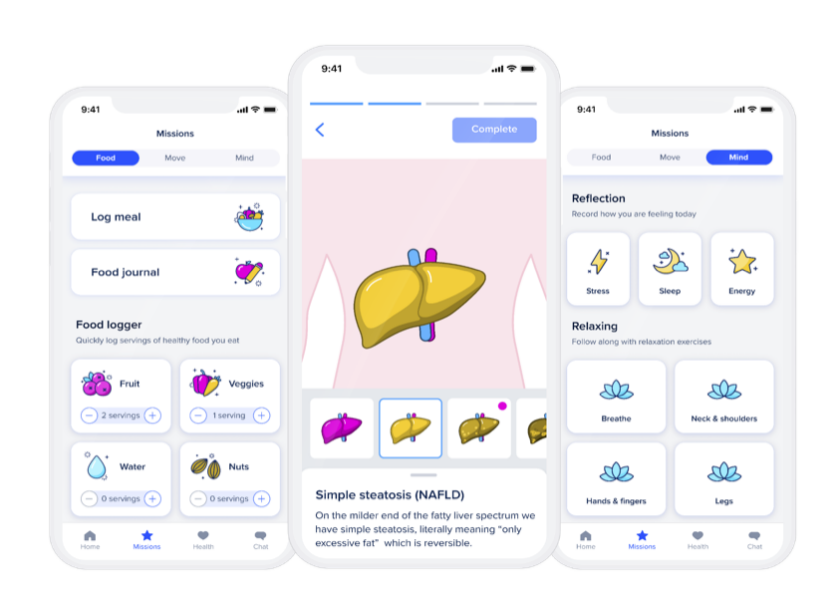
Example screens of the Sidekick app and the Sidekick-241 NAFLD program user interface. NAFLD: nonalcoholic fatty liver disease.

The SK-241 digital health program was developed by a multidisciplinary group of experts, including a clinical psychologist, nutritionist, behavioral scientists, medical doctors, and nurses at Sidekick Health. The primary focus of the program was to reduce participants’ daily dietary carbohydrate consumption and improve their overall nutrition quality in small, achievable, and sustainable steps (eg, reducing added sugars and processed foods, prioritizing protein, and increasing vegetable consumption). A secondary focus was to increase daily activity levels, improve sleep quality and reduce stress. The user interface with example screenshots from the program is shown in Figure 1.

The program included short daily missions (defined as in-app tasks for the participant to complete) aimed at increasing knowledge about NAFLD and NASH and its contributing factors and improving participants’ lifestyles for better metabolic health. The daily missions included watching short educational videos, reading brief informational content, logging meals and beverages by taking a photo of the meal, assessing on a sliding scale how healthy the meal was, and evaluating hunger and satiety before and after the meal. Other missions involved practicing mindfulness and meditation and logging daily energy levels, stress, and sleep quality. The app also provided participants with in-app health coach support (by a live person, not artificial intelligence), which provided weekly feedback on food logs and other in-app activities and opportunities for participants to ask questions as needed. The coaching element was active throughout the 12-week intervention period. Further details of the in-app content and missions are presented in Table 1 and Table S1 in Multimedia Appendix 1.

**Table 1:** The Sidekick-241 program content and descriptions of main missions.

Component	Description
Food journal	Participants were asked to log their meals at least 3 times per week each week. During week 2, individualized goals for gradually reducing carbohydrate intake throughout the program were set based on week 1 consumption.
Step counter	Participants could manually log their steps each day. Individualized goals for increasing steps were set for week 2 based on week 1 step counts.
QoL^a^ PROs^b^ (stress, sleep, energy)	Participants were prompted to log these measures 2 days per week on a 10-point visual-analog sliding scale.
Surveys	Questions about motivation levels, knowledge and attitudes relating to nutrition and physical activities were administered during weeks 1 and 2 and again during weeks 11 and 12. Questions about current food-related behaviors and potential NAFLD- or NASH-related symptoms were administered every 2 weeks.
Mindfulness	Participants were prompted to complete short mindfulness exercises regularly throughout the program and practice meditation 2 times per week from week 3 onwards.
Coaching	Feedback on weekly in-app activities was provided to participants, particularly on food logs and answers to the in-app surveys. Throughout the program, participants were also able to ask questions as needed and the coach would answer within 24 hours (weekends exempt).

^a^QoL: quality of life.

^b^PROs: patient-reported outcomes.

During the baseline visit, study staff assisted participants with downloading and installing the Sidekick app with the SK-241 program. A short web-based interview with the program’s health coach was offered to all participants during the first 2-3 weeks of the study to establish coach connection and accountability and to provide participants with an opportunity to ask questions. During the interview, the primary goals, main concepts, and the program’s approach to diet and weight management were explained. In addition, participants’ strengths and potential barriers to participation were discussed. 

### Outcome Measures and Covariates

Primary outcomes were the program’s feasibility and acceptability, as assessed by participant retention, engagement, and satisfaction after the 12-week study period. An active participant was defined as one completing at least 1 in-app mission or interacting with the health coach at least once per week. Retention was measured as the number of participants completing the 12-week program, which was defined as being active 9 of 12 weeks. Engagement was measured as the number of participants who were active during the whole 12-week period. Satisfaction with the program was assessed after program completion with the validated mHealth App Usability Questionnaire (MAUQ), which consists of 18 items and has a possible score of 0-7, with 7 being the highest potential score. The scoring can further be divided into 3 subscales reflecting ease of use (5 items), interface and satisfaction (7 items), and usefulness (6 items) [20]. In addition, detailed participant engagement with specific program features was analyzed.

Secondary outcomes were the program’s preliminary and potential clinical impact, as measured by weight loss, changes in liver fat, body composition, serum biomarkers, and other cardiometabolic risk factors (eg, blood pressure, waist and hip circumference, and step counts). Participants were assessed at baseline and at a 12-week follow-up visit for demographic information, anthropometric measures, medical history, medications, and adverse events. Liver fat content was measured and quantified at baseline and at 12 weeks using MRI-PDFF with a multiecho chemical shift–encoded gradient-echo sequence [21]. Body composition was assessed at baseline and at 12 weeks with a dual-energy X-ray absorptiometry [22]. Blood pressure was measured using an automatic blood pressure monitor. Blood samples were drawn at baseline and at the 12-week follow-up to measure complete blood count, alanine aminotransferase, aspartate aminotransferase, hemoglobin A_1c_ (HbA_1c_), fasting glucose and insulin for the homeostatic model assessment of insulin resistance (HOMA-IR), total cholesterol, high-density lipoprotein cholesterol, low-density lipoprotein cholesterol, triglycerides, and high-sensitivity C-reactive protein.

Participants were administered the following questionnaires via an electronic patient-reported outcome (PRO) system at baseline and at 12 weeks: the Depression, Anxiety and Stress Scale (DASS-21), the EuroQol-5 Dimension – 5-Level (EQ-5D-5L) index, and the 8-item Morisky Medication Adherence Scale (MMAS-8) [23-25].

For exploratory outcome analysis, study participants were divided into 2 groups depending on how engaged they were with the digital health program. Those using the app 5 or more days per week were defined as highly engaged compared with those using the app less than 5 days per week, and clinical outcomes were compared to assess a potential dose-response relationship.

### Statistical Analysis

As this is a feasibility study, a formal sample size calculation was not performed. The researchers aimed for 30-40 participants as this was considered a sufficiently sized sample to obtain information on practical aspects of participants’ recruitment, in-app engagement, retention, and rates of acceptance.

Changes in clinical assessments and PROs from baseline to postprogram were calculated as the mean and SD for approximately normally distributed variables (normality was analyzed with the Shapiro-Wilk test) or as the median and IQR for variables that did not satisfy normality criteria. Categorical data were calculated as frequencies and percentages. To compare baseline and postprogram outcomes, paired *t* tests were computed for approximately normally distributed data. In case the normality assumption was not met, nonparametric tests were computed (Wilcoxon signed-rank tests). Unless otherwise specified, all statistical tests were performed at the 5% (2-sided) significance level. Statistical analysis was performed in Stata (StataCorp) and R (version 4.0.3; R Foundation for Statistical Computing).

All enrolled participants were included in the full analysis set. Missing data were imputed using the last observation carried forward provided that the participant was enrolled in the study and at least one of two measurements (baseline or follow-up) was collected. Moreover, missing baseline measurements in waist circumference, hip circumference, and low-density lipoprotein cholesterol were imputed for 1 participant using the next observation carried backward. The complete case analysis set included participants who attended both the baseline visit and the 12-week follow-up visit.

### Ethical Considerations

This study was approved by the National Bioethics Committee of Iceland and the Data Protection Authority (22-075-Vl). All participants provided informed consent before being enrolled in the study. All data was deidentified and analyzed in accordance with institutional protocols. Participants were given the option of seeking reimbursement for travel expenses not exceeding US $150 in total; no other compensation was provided. The study was registered at ClinicalTrials.gov under the trial identifier NCT05426382.

## Results

### Participant Characteristics

**Figure 2:**
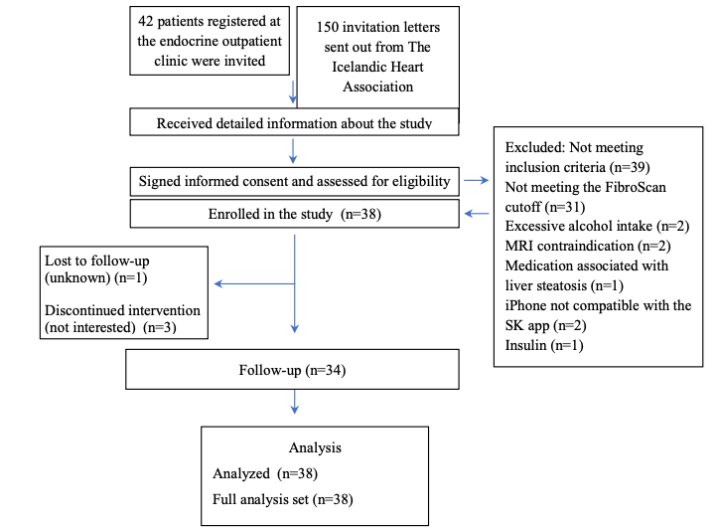
Flowchart of study participants. MRI: magnetic resonance imaging; SK: Sidekick.

After screening and enrollment, 38 individuals were eligible to participate in the study (Figure 2). The median age of the participants was 59.5 (IQR 46.3-68.8) years, 23 (61%) were women and all were White (Table 2). Of the 38 participants, 17 (45%) had a university degree, none smoked, 34 (90%) had obesity (BMI >30), 19 (50%) had type 2 DM, 27 (71%) had hypertension, 15 (40%) had hypercholesterolemia, and 11 (29%) had a history of cardiovascular disease. Other common comorbidities included hypothyroidism (n=11, 29%), polycystic ovary syndrome (n=4, 11%), and gout (n=2, 5%). In total, 45% (n=17) of participants reported taking antidiabetic medication, 79% (n=30) antihypertensive medication, 37% (n=14) antilipidemic medication, and 37% (n=14) hypothyroid medication. Additionally, 74% (n=28) reported taking other medications, such as proton-pump inhibitors (n=11, 29%), anticoagulants (n=11, 29%), antidepressants (n=8, 21%), vitamin B_12_ (n=5, 13%), nonsteroidal anti-inflammatory medication (n=4, 11%), and antihistamines (n=4, 11%). During the 12-week study period, 5 (13%) participants reported medication changes: 3 (8%) started new medications (one received antibiotics, one received calcium channel blockers, and one vitamin B_12_) and 2 (5%) reported dosage adjustments (one for diabetes medications and one for beta blockers and antidepressants).

**Table 2:** Baseline characteristics of the study participants.

Characteristics		Participants (n=38)
**Gender, n (%)**		
	Women	23 (61)
	Men	15 (39)
Age (years), median (IQR)		59.5 (46.3-68.8)
Ethnicity: White, n (%)		38 (100)
**Work status, n (%)**		
	Full-time	18 (47)
	Part-time	5 (13)
	Not in labor market	15 (39)
	Pension	11 (29)
	Disability	2 (5)
	Sick leave	1 (3)
	Unemployed	1 (3)
**Educational level, n (%)**		
	University degree	17 (45)
	Trades or vocational school or equivalent	12 (32)
	Primary education or less	6 (16)
	Secondary or matriculate	3 (8)
**Smoking status, n (%)**		
	Current smoker	0 (0)
	Never smoked	20 (53)
	Former smoker	18 (47)
**Comorbidities, n (%)**		
	Type 2 diabetes	19 (50)
	BMI >30	34 (89)
	Hypercholesterolemia	15 (39)
	Hypertension	27 (71)
	Cardiovascular disease	11 (29)
	Hypothyroidism	11 (29)
	Polycystic ovary disease	4 (11)
	Gout	2 (5)
	Other	23 (61)

### Retention and Engagement in the 12-Week Program

Of the 38 participants, 34 (89%) completed the 12-week program, 29 (76%) were engaged during the whole study period, and 22 (58%) were highly engaged (defined as visiting the app at least 5 days per week) (Table 3). Engagement and retention in the app were similar between those younger or older than 60 years and between men and women (data not shown). Participants were active in-app on a median of 81 (IQR 45.8-84.0) of 84 days or 6.8 (IQR 4.6-7.0) days per week on average and completed an average of 6.9 (SD 2.9) daily missions. Over the course of the study, the health coach sent an average 23.5 (SD 10.3) messages to participants, while participants sent and average of 15.5 (SD 12.4) messages to the coach, who responded within 1.2 (SD 0.9) days. The median MAUQ score was 6.3 (IQR 5.8-6.7) of 7, suggesting high satisfaction with the program among participants.

**Table 3:** Overall retention, engagement, and satisfaction.

Description					Values
**Primary endpoints**					
	Retention^a^, n (%)			34 (89)	
	Engagement^b^, n (%)			29 (76)	
	**Satisfaction^c^ median (IQR)**				
		MAUQ^d^ total score	6.3 (5.8-6.7)		
		Ease of use (mean of MAUQ items 1 to 5)	6.4 (5.6-6.8)		
		Interface and satisfaction (mean of MAUQ items 6 to 12)	6.3 (5.9-6.9)		
		Usefulness (mean of MAUQ items 13 to 18)	6.0 (5.5-6.7)		
**Exploratory engagement metrics**					
	Average active^e^ days per week (0-7), median (IQR)			6.8 (4.6-7.0)	
	Average total active days (0-84), median (IQR)			81 (45.8-84.0)	
	Average daily missions completed^f^, mean (SD)			6.9 (2.9)	
	Average daily missions assigned, mean (SD)			5.7 (0.49)	
	Participants who were active >5 days every week, n (%)			22 (58)	
	Average number of messages sent by participants, mean (SD)			15.5 (12.4)	
	Average number of messages received by participants, mean (SD)			23.5 (10.3)	

^a^Retention was defined as participants who completed the program, being active for 9 of 12 weeks. Being active was defined as completing at least 1 in-app mission or interacting at least once in that week with the health coach.

^b^Engagement was defined as participants who were active for the entire study period.

^c^Satisfaction was measured using the MAUQ.

^d^MAUQ: mHealth App Usability Questionnaire.

^e^An active day was defined as a day in which the participant completed at least 1 in-app mission or interacted with the coach.

^f^The participants receive daily assigned missions but also had the opportunity to complete additional missions within the app, thereby surpassing the number of assigned missions.

### Metabolic Parameters

The mean weight loss was 3.5 (SD 3.7) kg (*P*<.001), or 3.2% (SD 3.4%) (Table 4). The median body fat percentage changed from 46.6% (IQR 39.4%-52.4%) to 44.3% (IQR 37.8%-52.2%) (*P*<.001) and the mean fat mass from 50.3 (SD 13.8) kg to 48.1 (SD 14.5) kg (*P*<.001). These improvements in body composition were accompanied by reduced MRI-PDFF liver fat values: in the full analysis set (n=38), the mean liver fat percentage significantly decreased from 12.3% (SD 7.1%) to 10.1% (SD 6.5%; *P*<.001), representing a mean relative change of 19.4% (SD 23.9%) (Table 4). In the complete case analysis set (n=34), mean liver fat was reduced from 12.4% (SD 6.9%) to 9.9% (SD 6.3%; *P*<.001) with a corresponding mean relative change of 21.6% (SD 24.2%).

**Table 4:** Differences in anthropometric, biochemical, and clinical measurements at baseline and after 12 weeks for the full analysis set (n=38).

			Baseline		Week 12		Change from baseline to week 12		*P* value
**Anthropometry**									
	Weight (kg), mean (SD)	110.0 (18.5)		106.5 (18.4)		3.5 (3.7)		<.001^a^	
	Relative percentage weight change^b^, mean (SD)	N/A^c^		N/A		3.2 (3.4)		N/A	
	BMI (kg/m^2^), mean (SD)	37.6 (5.8)		36.4 (5.8)		1.2 (1.3)		<.001^a^	
	Waist circumference (cm), mean (SD)	123.8 (12.2)		119.9 (12.2)		4.0 (5.1)		<.001^a^	
	Hip circumference (cm), mean (SD)	125.1 (14.0)		123.2 (13.3)		1.8 (0.0 to 4.9)		.01^d^	
	Waist to hip ratio, median (IQR)	1.00 (0.95 to 1.03)		0.99 (0.92 to 1.03)		0.00 (–0.01 to 0.03)		.09^d^	
**Liver assessment**									
	Liver fat MRI-PDFF^e^ (%), mean (SD)	12.3 (7.1)		10.1 (6.5)		2.2 (2.9)		<.001^a^	
	Liver fat MRI-PDFF relative change^b^ (%), mean (SD)	N/A		N/A		19.4 (23.9)		N/A	
	Liver stiffness measure (kPa), median (IQR)	6.4 (5.2 to 9.6)		6.6 (5.3 to 8.4)		0.2 (–0.3 to 1.6)		.11^d^	
	CAP^f^ score (dB/m), mean (SD)	343.6 (34.8)		310.3 (47.2)		33.3 (39.7)		<.001^a^	
**Body composition^g^**									
	Total body region fat (%), median (IQR)	46.6 (39.4 to 52.4)		44.3 (37.8 to 52.2)		0.9 (1.4)^b^		<.001^a^	
	Fat mass (kg), mean (SD)	50.3 (13.8)		48.1 (14.5)		2.2 (2.7)		<.001^a^	
	Lean mass (kg), mean (SD)	56.3 (10.1)		55.6 (9.7)		0.7 (1.7)		.008^a ^	
**Blood pressure (mmHg), mean (SD)**									
	Systolic	141.4 (17.1)		135.4 (17.3)		6.0 (13.5)		0.009^a^	
	Diastolic	83.6 (7.4)		82.5 (7.4)		1.2 (7.7)		.36^a^	
**Biochemical measures**									
	HbA_1c_^h^ (mmol/mol), median (IQR)	60.0 (56.0 to 66.8)		60.0 (54.3 to 64.0)		0.5 (–0.7 to 3.8)		.03^d^	
	S-glucose^i^ (mmol/L), median (IQR)	6.2 (5.3 to 7.4)		6.3 (5.4 to 6.9)		0.0 (–0.3 to 0.4)		.64^d^	
	S-insulin^j^ (µU/ml), median (IQR)	21.1 (16.4 to 27.9)		19.0 (13.0 to 25.0)		3.2 (0.0 to 5.4)		.003^d^	
	HOMA-IR^k^ (mmol/L), median (IQR)	5.8 (4.3 to 8.4)		4.8 (3.6 to 7.2)		0.4 (–0.2 to 2.1)		.02^d^	
	Total cholesterol (mmol/L), mean (SD)	4.9 (1.3)		4.8 (1.2)		0.0 (–0.2 to 0.2)		>.99^d^	
	LDL-C^l^ (mmol/L), mean (SD)	2.9 (1.1)		2.9 (1.1)		–0.1 (–0.3 to 0.1)		.18^d^	
	HDL-C^m^ (mmol/L), mean (SD)	1.11 (0.23)		1.12 (0.19)		–0.01 (0.12)		.56^a^	
	Triglycerides (mmol/L), median (IQR)	1.88 (1.35 to 2.45)		1.68 (1.21 to 1.90)		0.14 (0.00 to 0.47)		.003^d^	
	hs-CRP^n^ (mg/L), median (IQR)	3.0 (1.2 to 5.2)		2.5 (1.1 to 3.9)		0.1 (–0.1 to 0.7)		.14^d^	
	ALAT^o^ (IU/L), median (IQR)	21.4 (18.2 to 30.2)		23.2 (18.4 to 32.0)		0.0 (–6.8 to 2.8)		.37^d^	
	ASAT^p^ (IU/L), median (IQR)	20.8 (17.9 to 24.8)		22.3 (18.0 to 25.5)		0.4 (–2.5 to 2.5)		.53^d^	
	FIB-4^q^ Index, median (IQR)	1.08 (0.78 to 1.34)		1.08 (0.75 to 1.21)		0.01 (–0.06 to 0.07)		.58^d^	

^a^Analyzed with a paired *t* test.

^b^Percentage change calculated as the average over individual relative changes.

^c^N/A: not applicable.

^d^Analyzed with a Wilcoxon signed-rank test.

^e^MRI-PDFF: magnetic resonance imaging proton density fat fraction.

^f^CAP: controlled attenuation parameter.

^g^Measured by dual-energy ray absorptiometry.

^h^HbA_1c_: glycated hemoglobin A_1c_.

^i^s-glucose: Serum glucose.

^j^s-insulin: Serum insulin.

^k^HOMA-IR: homeostatic model assessment of insulin resistance.

^l^LDL-C: low-density lipoprotein cholesterol.

^m^HDL-C: high-density lipoprotein cholesterol.

^n^hs-CRP high-sensitivity C-reactive protein.

^o^ALAT: alanine aminotransferase.

^p^ASAT: aspartate aminotransferase.

^q^FIB-4: index for liver fibrosis.

During the study, the distribution of steatosis levels changed. At baseline, the 10%-15% liver steatosis category had the highest frequency with 32% (n=12) of participants. At follow-up, the 5%-10% liver steatosis category had the highest frequency with 34% (n=13) of participants (Table 5). We additionally found a significant correlation between weight loss and absolute (*r*=0.48, *P*=.004) and relative (*r*=0.72, *P*<.001) liver fat changes measured by MRI-PDFF.

According to the FIB-4 data, 4 of the 38 participants were classified as having a high risk of fibrosis at baseline, of which 1 individual regressed to intermediate risk at the 12-week follow-up visit. Most participants (n=27, 71%) had a low risk of fibrosis at baseline according to the FIB-4. At the 12-week follow-up, this percentage had gone up to 79% (n=30).

Mean systolic blood pressure significantly decreased by 6.0 (SD 13.5) mmHg (*P*=.009), and this was not explained by changes in medication or medication adherence (Table 4). There was no significant difference in diastolic blood pressure.

Participants recorded on average 3085 (SD 2246) daily steps in the first week and 4664 (SD 3780) daily steps in the last week, representing a significant increase of 1579 steps per day *(P*=.02).

While participants’ average baseline fasting insulin and HOMA-IR levels indicated insulin resistance, we found a significant decrease in serum insulin levels (median 3.2, IQR 0.0-5.4 µU/ml; *P*=.003), HOMA-IR levels (median 0.4, IQR –0.2 to 2.1 mmol/L; *P*=.02), and HbA_1c_ levels (median 0.5, IQR –0.7 to 3.8 mmol/mol; *P*=.03) (Table 4), suggesting improved glycemic control. In addition, triglyceride levels significantly decreased by a median of 0.14 (IQR 0.00-0.47) mmol/L (*P*=.003), and median high-sensitivity C-reactive protein levels decreased from 3.0 (IQR 1.2-5.2) mg/L to 2.5 (IQR 1.1-3.9) mg/L (*P*=.14) (Table 4), representing improvements in those cardiovascular risk factors.

We did not find any significant change in cholesterol levels, nor any significant changes in PRO scores of health-related quality of life, mental health, or medication adherence from preprogram to postprogram (Table 6).

**Table 5:** Distributions of liver fat percentage categories based on magnetic resonance imaging proton density fat fraction liver fat values at baseline and at the 12-week follow-up (n=38). All participants with >5% liver fat at baseline had stage 1 steatosis according to the standardized Nonalcoholic Steatohepatitis Clinical Research Network histologic scoring system for nonalcoholic fatty liver disease [26].

Liver fat category (%)^a^	Participants at baseline, n	Participants at week 12, n
<5	6	9
5-10	9	13
10-15	12	7
15-20	5	5
20-25	4	3
25-30	2	1

^a^Limits for the presented ranges correspond to values greater than or equal to for lower limits and less than for upper limits.

**Table 6:** Differences in patient reported outcomes (PROs) at baseline and after 12 weeks for the full analysis set (n=38).

PROs		Baseline	Week 12	Change from baseline to week 12	*P* value^a^
EQ-5D-5L index^b^		0.8 (0.7 to 0.9)	0.9 (0.8 to 1.0)	0.0 (–0.1 to 0.0)	.36
**DASS-21^c^, median (IQR)**					
	Total score (0 to 56)	5.5 (2.0 to 13.0)	6.0 (2.0 to 11.0)	0.0 (–2.0 to 2.0)	.95
	Depression score	1.0 (0.3 to 4.5)	1.0 (0.0 to 3.0)	0.0 (0.0 to 1.0)	—^d^
	Anxiety score	1.0 (0.0 to 2.0)	1.0 (0.0 to 2.0)	0.0 (–0.7 to 1.0)	—
	Stress score	3.0 (1.0 to 6.0)	3.0 (0.3 to 6.0)	0.0 (–1.0 to 1.0)	—
**MMAS-8^e^**					
	Total score (0 to 8)	7.0 (6.8 to 8.0)	7.0 (6.8 to 8.0)	0.0 (–0.7 to 0.0)	.68
	High adherence (=8), n (%)	14 (37)	15 (39)	N/A^f^	—
	Moderate adherence (6 to 7), n (%)	15 (39)	15 (39)	N/A	—
	Low adherence (<6), n (%)	9 (24)	8 (21)	N/A	—

^a^Analyzed with Wilcoxon signed-rank test.

^b^EQ-5D-5L index: EuroQol-5 Dimension – 5-Level index.

^c^DASS-21: Depression, Anxiety, and Stress Scale - 21 Items.

^d^Not available.

^e^MMAS-8: 8-item Morisky Medication Adherence Scale.

^f^N/A: not applicable.

### Associations Between App Engagement and Clinical Outcomes

An exploratory analysis was performed to assess the relationship between participants’ in-app activity and their clinical outcomes. We found that participants who were highly engaged (visited the app at least 5 days per week) had greater weight loss and liver fat reduction (Table S2 in Multimedia Appendix 1) compared with those who were less engaged. In a complete case analysis, participants who were highly engaged (n=22) lost on average 5.1 (SD 3.8) kg and achieved a 27.5% relative reduction in liver fat, while those who were active on fewer than 5 days a week (n=12) lost on average 1.8 (SD 2.2) kg and achieved 10.8% relative reduction in liver fat. Moreover, highly engaged participants were significantly more likely to achieve a relative weight loss of at least 3% (*P*=.001) or 5% (*P*=.02) compared with those who were less engaged (Fisher exact tests). Taken together, these results suggest that higher engagement with the digital program may be associated with improved metabolic health.

### Adverse Events

In total, 9 adverse events were reported, all of which were of mild to moderate intensity with no serious adverse events (Table S3 in Multimedia Appendix 1). No adverse events were considered to have a causal relationship to the digital health program, as assessed by the primary investigator. 

## Discussion

### Principal Findings

This study demonstrated that the 12-week-long digital health program, SK-241, was feasible given its high retention, engagement, and satisfaction among people with NAFLD. Cardiometabolic health and liver-specific outcomes improved over the 12-week study period with a significant weight loss and reductions in fat mass, liver fat, systolic blood pressure, triglycerides, insulin, and HbA_1c_ levels.

Digital behavioral programs can be effective at targeting weight loss among people with chronic conditions [27]. Increasing evidence shows that programs—whether digital or face-to-face—with a holistic approach can also be effective for people with NAFLD, where weight loss is a major component of disease management. A recent randomized controlled study from Singapore including 108 adults with NAFLD randomized either to lifestyle advice by a trained nurse or using a lifestyle mobile app in addition to receiving advice by a dietitian showed that the mobile app group had a 5-fold higher likelihood of achieving ≥5% weight loss compared with the control group at 6 months [28].

Previous studies suggest that digital solutions can be as effective as face-to-face behavioral change programs, but engagement with the digital program is an important component of efficacy [29]. Indeed, an important finding of this study was the correlation between participants’ in-app engagement and their clinical outcomes; this shows that maintaining engagement and interest is key to reaching the desired clinical improvements. Program engagement may be influenced by several factors, such as recruitment methods, participant characteristics, app design, and the level of support, such as coaching [30,31]. Coaching in particular may be essential to drive engagement as it encourages accountability and may increase motivation [30]. The regular contact that participants had with the coach in the SK-241 program may have contributed to the low attrition and high engagement in this study. Should larger implementation of this intervention take place, then coaching would be an integral part, at least in the initial stages of the program.

Lifestyle interventions consisting of diet, exercise, and weight loss are recommended to individuals with NAFLD according to treatment guidelines [2]. The primary driver of NAFLD is overnutrition, which causes expansion of adipose deposits and macrophage infiltration into the visceral adipose tissue, creating a proinflammatory state that promotes insulin resistance [32,33]. The resulting imbalance in lipid metabolism leads to the formation of lipotoxic lipids that contribute to cellular stress, including oxidative stress, inflammasome activation, and apoptotic cell death [34,35]. Central obesity is also an important driver of insulin resistance and proinflammatory signaling [36]. In this 12-week study, the mean waist circumference was significantly reduced by 4.0 cm, and body weight by 3.2% on average; these are encouraging results, considering that a 3%-5% weight loss can lead to a reduction in hepatic steatosis [37]. In addition, we found a correlation between weight loss and MRI-PDFF liver fat fraction changes. This is in line with a previous report of greater weight loss leading to more significant improvements in liver histopathology, and studies have shown that a ≥30% relative decline in liver fat by MRI-PDFF is associated with histopathological improvements in NASH [38-40]. Participants in this study were able to decrease their waist circumference and body weight and had an average of around a 20% relative reduction in liver fat by MRI-PDFF, with a subset of participants achieving a 30% relative decline, which might lead to improved NAFLD and NASH histopathology.

Despite the risk of progressive liver disease, the leading cause of death in people with NAFLD is cardiovascular disease [10]. This is likely due to risk factors that are shared between NAFLD and cardiovascular diseases, although it is unclear to what extent NAFLD has a direct causative role in the development of cardiovascular disease [41]. Therefore, it was important to see significant improvement in cardiovascular risk factors in our study, such as a decrease in systolic blood pressure, triglycerides, insulin, and HbA_1c_. The increased physical activity in our study as measured by the in-app step counter and the correlation between in-app activity and weight loss suggest that the digital program may successfully engage participants in behaviors that lead to more weight loss, which in turn may hypothetically improve liver function and glycemic control. Regular tracking of meals and physical activity and completing the in-app PROs may help people become more aware of their habits, while the education and the coach’s feedback and support may give them the necessary tools to change their behaviors. We did not find any significant changes in the PRO scores of health-related quality of life and mental health, which was most likely due to the short duration of the study and the small size of the cohort.

Furthermore, studies have shown that health care utilization and expenditure are particularly high among people with NAFLD and NASH [42,43]. Therefore, there is a great need for early identification and effective management of people with NAFLD to minimize the comorbidity burden and health care costs.

The fibrosis risk among study participants was assessed both with a VCTE FibroScan LSM and by calculating the FIB-4 index score from participants’ age and the serum alanine aminotransferase, aspartate aminotransferase, and platelet count. The results indicated that a few participants had an intermediate to high risk of having liver fibrosis (data not shown) and could be referred to as probable patients with NASH, thereby suggesting that the digital health program might be feasible for individuals with NASH in addition to those with NAFLD. However, both of these measurements have their limitations and need to be interpreted cautiously. VCTE can rule out advanced fibrosis but often leads to false positive results in NAFLD, while the FIB-4 score might overestimate fibrosis in populations older than 65 years and is considered to have a low positive predictive value for identifying advanced fibrosis [44,45].

### Strengths and Limitations

A strength of this study was the high engagement and completion rate, as these are well known issues of digital health programs [46]. In addition, the holistic nature of the program, developed by a multidisciplinary team of experts and focused on multiple aspects of participants’ lifestyle, combined with the regular support provided by the coach, can be considered a strength. A further strength was the length of the program, which allowed sufficient time to assess meaningful changes in engagement and clinical outcomes.

Limitations of this study included the single-arm design, which limits the interpretation and generalizability of our findings. The lack of a control group made it difficult to directly infer the clinical benefit of digital program, thus the secondary outcomes relating to clinical efficacy should be interpreted with caution. The observed clinical improvements should also be interpreted in context with the short duration of the health program, as sustaining improvements can be challenging after short-term behavioral interventions. It should also be acknowledged that a seasonal increase in activity levels may have contributed to the observed changes, as the study began in early summer when people tend to be more physically active. Furthermore, all the participants were White and around 50% had a relatively high education level. Higher education level has been associated with a lower burden of traditional cardiovascular risk factors [47]. Previous studies have shown an association between socioeconomic status and NAFLD, where poverty seems to be a risk factor for developing NAFLD independent of other known risk factors, such as type 2 DM and obesity, and food insecurity is associated with developing NAFLD and advanced fibrosis [48]. Education and smoking status may have affected engagement with the digital health program and, therefore, the generalizability of these results to a wider population may be limited and future trials should recruit a more diverse group of participants to assess the efficacy of the program [49]. 

### Conclusions

The 12-week-long digital health program was feasible for individuals with NAFLD, showing high user engagement, retention, and satisfaction. Improved liver-specific and cardiometabolic health was observed and more engaged participants showed greater improvements. This NAFLD digital health program could provide a new tool to improve health outcomes in people with NAFLD.

## Appendix

Multimedia Appendix 1 Supplementary tables.

## Abbreviations

CAP: controlled attenuation parameter
DASS-21: Depression, Anxiety, Stress Scale, 21 Items
DM: diabetes mellitus
EQ-5D-5L index: EuroQol-5 Dimension – 5-Level index
HbA1c: hemoglobin A1c
HOMA-IR: homeostatic model assessment for insulin resistance
LSM: liver stiffness measurement
MAUQ: mHealth App Usability Questionnaire
MMAS-8: 8-item Morisky Medication Adherence Scale
MRI-PDFF: magnetic resonance imaging proton density fat fraction
NAFLD: nonalcoholic fatty liver disease
NASH: nonalcoholic steatohepatitis
ppt: percentage points
PRO: patient-reported outcome
s-insulin: Serum insulin
Total-C: total cholesterol
VCTE: vibration-controlled transient elastography

## Data Availability

The data sets generated or analyzed during this study are not publicly available due to restrictions in the informed consent form. Additional summary statistics will be provided upon on reasonable request.
