# Supplementary material for: User Engagement, Acceptability, and Clinical Markers in a Digital Health Program for Nonalcoholic Fatty Liver Disease: Prospective, Single-Arm Feasibility Study
Source: JMIR Cardio. 2024 Feb 15;8:e52576. doi: 10.2196/52576 (PMC10905363; doi:10.2196/52576)
Supplement: Multimedia Appendix 1 [file cardio_v8i1e52576_app1.docx]

**Supplementary tables**

**Table S1.** Weekly educational content topics over the 12-week in-app program.

| **Week number** | **Content topics** |
| --- | --- |
| 1 - Introduction | Introduction to the program; NAFLD/NASH basic education, baseline logs for activity and daily food and beverage consumption. |
| 2 - Sugars | The effect of sugar on the body while integrating the goals, plans, and the general behavior change process. |
| 3 - Mindfulness | Reducing sugar and sedentary behavior, increasing physical activity, introducing stress and mind in the app. |
| 4 - Metabolic health | Educate on the spectrum of metabolic health, offer supporting tips for prolonging the overnight fast, introduce a framework of portion control, hunger and satiety awareness, and mindful eating. |
| 5 - Proteins | Meal planning, importance of proteins over carbs, hunger and satiety, macronutrient groups. |
| 6 - Carbohydrates | Create a purpose, understanding and motivation for changed nutrition; introduce carbs as a macronutrient, “good” and “bad” carbs and relevance to NAFLD; introduction to mindful eating and craving triggers. |
| 7 - Fats | Introduce dietary fats and relevance to NAFLD; “good” fats and “bad” fats; importance of Vitamin D, introduction to the microbiome. |
| 8 - Sleep | Introduce the importance of sleep and tips on improving sleep. |
| 9 - Physical activity | The benefits of daily physical activity and reducing sedentary behavior. Discussion about values and living life with purpose. |
| 10 - Food-related skills | More information on previous topics (e.g. meal planning). |
| 11-  Managing difficult emotions | Psychological tools for dealing with difficulties. |
| 12 - Recap and maintenance of new habits | Program highlights recap, the importance of recognizing small wins, the process of changing behavior, recognizing the effort. |

**Table S2.** Subgroup analysis of changes from baseline to week 12 for the clinical outcomes split by engagement status (active ≥ 5 days/week or <5 days/week). Analyzed for the full analysis set (FAS) and the complete case set (CC)

|  | FAS | | CC | |
| --- | --- | --- | --- | --- |
|  | Active ≥ 5 days/week (n=22) | Active <5 days/week  (n=16) | Active ≥ 5 days/week (n=22) | Active <5 days/week (n=12) |
| Weight loss, mean kg (SD) | -5.1 (3.8) | -1.4 (2.1) | -5.1 (3.8) | -1.8 (2.2) |
| Relative percentage change in weight, mean (SD) | -4.8 (3.5) | -1.1 (1.8) | -4.8 (3.5) | -1.5 (1.9) |
| Absolute change in liver fat percentage, mean percentage points (SD) | -3.1 (2.6) | -1.0 (2.9) | -3.1 (2.6) | -1.3 (3.3) |
| Relative percentage change in liver fat, mean (SD) | -27.5 (21.4) | -8.1 (23.0) | -27.5 (21.4) | -10.8 (26.2) |

**Table S3.** Occurrence of adverse events during the study period.

| Adverse event | No. of total events | No. of events by severity | |
| --- | --- | --- | --- |
|  |  | Mild | Moderate |
| Total | 9 | 8 | 1 |
| Knee pain | 1 | 1 | - |
| Sleep apnea | 1 | - | 1 |
| Infection | 1 | 1 | - |
| Flu | 1 | 1 | - |
| Common cold | 3 | 3 | - |
| Fall | 1 | 1 | - |
| Worsening depression | 1 | 1 | - |
